# Investigation of potential collider bias in estimating the association between long-term exposure to air pollution and COVID-19 mortality

**DOI:** 10.1097/EE9.0000000000000394

**Published:** 2025-04-28

**Authors:** Jiawei Zhang, Zorana Jovanovic Andersen, George Maria Napolitano, Youn-Hee Lim

**Affiliations:** a Section of Environmental Health, Department of Public Health, University of Copenhagen, Copenhagen, Denmark

**Keywords:** Air pollution, COVID-19, Collider bias, Mortality

## Abstract

**Background:**

Patient-based cohorts were frequently used to investigate air pollution-related coronavirus disease 2019 (COVID-19) evidence, which can be subject to collider bias. However, this bias has not been explored. We aimed to quantify and adjust the collider bias by limiting study population to patients with COVID-19 when estimating the association between long-term exposure to air pollution (LTAP) and COVID-19 mortality.

**Methods:**

In a register-based cohort study including 3,721,813 residents aged 30 or older in Denmark, we followed them from 1 March 2020 to 26 April 2021. We estimated the hazard ratios of COVID-19 mortality associated with LTAP. We calculated the relative hazard ratios (RHR) by comparing the hazard ratios of COVID-19 mortality among the general population and patients (infected or hospitalized) to quantify the impact of collider bias, and further applied inverse probability weighting (IPW) to adjust the potential collider bias.

**Result:**

We detected 138,742 positive for SARS-CoV-2, 11,270 COVID-19 hospitalizations, and 2557 deaths from COVID-19 during the study period. Although the demographic and socioeconomic characteristics differed among the three populations (general population, infected individuals, and hospitalized patients), infected and hospitalized patients experienced higher air pollution exposure compared with the general population. We observed greater associations of exposure to air pollution with COVID-19 mortality in the general population compared with the COVID-19 infected and hospitalized patients, with RHR of 0.88 (0.82, 0.95) and 0.85 (0.74, 0.97) per 0.55 µg/m^3^ increase in fine particulate matter (PM_2.5_) when we limited to infected and hospitalized patients, respectively. Similar association was also observed with nitrogen dioxide exposure. After applying IPW, we observed moderate increase in the estimated association, with RHR altered to null.

**Conclusion:**

Our findings suggest that associations between LTAP and COVID-19 mortality are likely underestimated in patient-based cohorts due to potential collider bias, while IPW could be a useful tool to adjust such bias.

**What this study adds**

This study showed how collider bias can distort findings in patient-based cohorts, leading to potentially misleading conclusions about the association between long-term air pollution and COVID-19 mortality. Specifically, we found that the association between long-term air pollution and COVID-19 mortality is likely underestimated in patient-based cohorts due to collider bias. By applying relative hazard ratios and inverse probability weighting, we provide a quantitative assessment of how selection mechanisms influence effect estimates and offer a methodological approach to address collider bias inherent in patient-based cohorts. These findings enhance the interpretation of epidemiological results and provide valuable insights for future research.

## Introduction

Air pollution has gained increasing attention as a contributing factor to severe acute respiratory syndrome coronavirus 2 (SARS-CoV-2) infection and coronavirus disease 2019 (COVID-19) severity.^1^ This interest is underscored by the recognition that air pollution may exacerbate respiratory infections, and is linked with pneumonia and respiratory mortality.^2–4^ However, due to the scarcity of studies in general populations with comprehensive SARS-CoV-2 test results, most of the evidence in examining the association between exposure to air pollution and COVID-19 severity has been inferred from “patient-based” samples. These samples comprise individuals who tested positive for SARS-CoV-2 or were admitted to hospital.^5^ The patients in the cohorts were subsequently monitored for hospital admissions (in the case of positive cases) or mortality.

**Figure 1.**
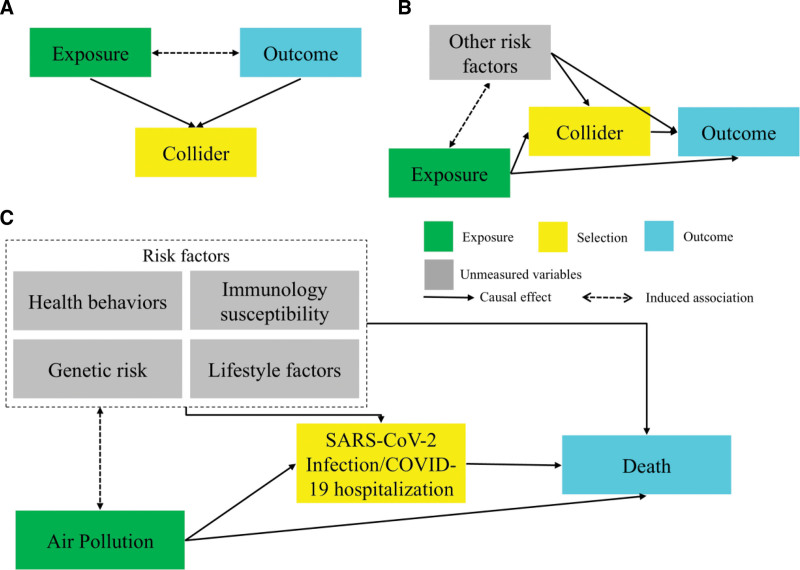
Conceptualized directed acyclic graph for the situation collider bias is induced.

Such methodologies employing patient samples may be notably vulnerable to collider bias,^6^ a phenomenon that arises when conditioning on a common effect of two otherwise unrelated factors, inadvertently creating a spurious statistical association between them. The simplest scenario of collider bias is depicted in Figure 1A as a directed acyclic graph, where both the exposure and outcome are causally independent in the general population but both influence a third variable, the collider, which is conditioned upon during analysis. Conditioning on the collider induces an artificial association between the exposure and the outcome, even in the absence of a true causal relationship.

Figure 1B represents the general case of patient-based cohorts, where conditioning on an intermediate variable (e.g., hospitalization) in the disease prognosis process introduces statistical intercorrelation among all the risk factors for that intermediate variable. For instance, in prognosis studies, participants often already have underlying conditions such as cancer, diabetes, or COVID-19. These conditions are typically associated with multiple independent risk factors. However, conditioning on hospitalization, as an intermediate variable, would create spurious associations among these independent risk factors, complicating the disentanglement of true causal relationships between the exposure, the intermediate variable, and the outcome. This challenge is common across many observational studies.

In the context of COVID-19 research, growing evidence has established independent links between long-term exposure to air pollution, genetic predisposition, lifestyle factors, and underlying health conditions with an increased risk of SARS-CoV-2 infection and subsequent hospitalization.^5–7^ As illustrated in Figure 1C, conducting a study within a population of SARS-CoV-2-infected or hospitalized patients introduces collider bias. Conditioning on infection or hospitalization creates spurious associations between air pollution and other risk factors, such as genetic and lifestyle factors, which are otherwise independent of air pollution in the general population. These spurious associations cause the risk factors to act as confounders in the relationship between air pollution and mortality. Consequently, this can lead to misinterpretation of the true causal relationship between air pollution (the exposure) and COVID-19 severity or mortality (the outcome).

While collider bias has been well-discussed and documented in clinical epidemiology,^5,6^ there is a notable gap in the environmental epidemiology literature regarding this statistical issue. This disparity might partially explain why the impact of collider bias on assessing the association between long-term exposure to air pollution and COVID-19 mortality has not been empirically explored to a significant extent. Although explicitly validated methodologies for assessing collider bias are limited, a long-standing body of literature supports the use of relative estimates to evaluate selection bias, of which collider bias is a specific form. Criqui et al.^8,9^ and Kleinbaum et al.^10^ were among the first to propose relative estimates as a quantitative measure for selection bias. Greenland later emphasized their utility in quantifying bias introduced by stratification on variables influenced by both exposure and outcome (e.g., colliders).^11^ This methodological framework has since been applied to assess various forms of selection bias, including nonresponse bias,^12,13^ self-selection bias,^14^ and loss-to-follow-up bias.^15^

However, quantifying collider bias is only the first step; addressing it requires robust methodological approaches. One such approach is inverse probability weighting (IPW), which has been widely used to adjust for selection bias in epidemiological studies.^6,13^ IPW works by assigning weights to individuals based on their probability of being included in the sample, thereby creating a pseudo-population that approximates the target population. Recently, IPW has been proposed as a method to address collider bias, particularly in studies where the collider (e.g., COVID-19 infection) is the condition under which data are collected.^16–18^ By weighting individuals based on their probability of being infected, inferred from external data or representative sample and estimated conditional on covariates such as air pollution exposure and contextual factors, IPW is suggested to help mitigate the bias introduced by conditioning on the collider.

We have previously reported a positive association of long-term exposure to air pollution (fine particulate matter [PM_2.5_] and nitrogen dioxide [NO_2_]) with COVID-19 mortality in a population-based nationwide cohort in Denmark.^19^ We now extend these results and aim to address this gap by providing a more comprehensive understanding of the impact of collider bias that might arise from using SARS-CoV-2-infected or -hospitalized patients as the baseline population when estimating associations with COVID-19 mortality in the Danish population, and to contribute to the development of robust methods for quantifying and addressing this issue in future research. To achieve this, we calculate the relative hazard ratios (RHRs) based on the estimated HRs under both conditional and unconditional analyses and used bootstrapping techniques to calculate the 95% confidence intervals (CIs) to quantify the distortion introduced by the collider bias. Furthermore, we apply IPW to adjust for this bias, offering a novel contribution to the environmental epidemiology literature by providing a more nuanced understanding of the relationship between air pollution and COVID-19 severity.

## Methods

In this population-based cohort study, we used data from air pollution and COVID-19 study in Denmark (AIRCODEN), including all Danish residents aged 30 years or above on 1 March 2020 (Baseline), and had lived in Denmark for at least 1 year.^19^ Each resident in Denmark has a unique personal identification number, which is further used to link with the National Socioeconomic registers at Denmark Statistics to obtain comprehensive demographic, individual, and area-level socioeconomic status (SES) information.^20,21^ All individuals were followed from baseline until the date of death, emigration, or end of the study period, 26 April 2021. We retrieved individual-level information on SARS-CoV-2 testing by reverse transcription polymerase chain reaction (RT-PCR), subsequent hospitalizations, and mortality data, from the Danish National COVID-19 Surveillance System.^22^ In Denmark, RT-PCR testing for SARS-CoV-2 was offered free of charge to all residents throughout the study period. Antigen tests only started to emerge at the end of the follow-up, and according to national recommendations, a positive antigen test must be confirmed with an RT-PCR test to be registered in the National COVID-19 Surveillance System. COVID-19 mortality, our primary outcome, was defined as death from any cause occurring within 30 days of the first positive SARS-CoV-2 test. This definition is aligned with the Danish National COVID-19 surveillance system, ensuring alignment with national reporting standards.

Two subcohorts were further defined by including (1) infected population: all participants since their first-ever RT-PCR positive test for SARS-CoV-2 (subcohort I) and (2) hospitalized patients: all the patients since any hospitalization that occurs within 14 days of the first sample with a positive SARS-CoV-2 RT-PCR test (subcohort II).

Annual exposure to PM_2.5_ and NO_2_ in 2019 was estimated using a validated Danish DEHM/UBM model at a spatial resolution of 1 km × 1 km.^23^ Individual residential address histories for 2019 were retrieved from the Danish Civil Registration System, which records all residential addresses for all residents of Denmark, along with the dates of any address changes. For individuals with multiple addresses during the study period, we calculated time-weighted mean exposure based on the duration of time spent at each address. There was no restriction on the number of address changes, and we accounted for all address updates before the study baseline.

## Statistical analysis

### Quantification of collider bias

In our study, we applied the RHR to assess the potential impact of collider bias, which arises when conditioning on a variable that is influenced by both the exposure (air pollution) and other independent risk factors for the outcome (COVID-19 mortality). The RHR compares hazard ratios from the general population (unconditional analysis) with those derived from a subset population defined by hospitalization or infection (conditional analysis).

Specifically, we used Cox proportional hazard models to estimate the association of long-term exposure to PM_2.5_ and NO_2_ with COVID-19 mortality. We first applied the regression model to the general population to estimate the hazard ratio ($HR_{Tot}$) without conditioning on the collider. This analysis aimed to obtain the association in the total population, unaffected by collider bias, with the main finding being reported in our previous study.^19^ The same regression model was then applied to a subset population defined by hospitalization or infection (conditional analysis) to estimate the hazard ratio ($HR_{Sub}$) conditioning on the collider. Finally, we quantified the collider bias as the RHR, calculated using the formula:


$$\begin{array}{l} RHR=\frac{HR_{Sub}}{HR_{Tot}} \end{array}$$

in which, a significant deviation of the RHR from 1 indicates potential distortion in the exposure–outcome relationship caused by conditioning on the collider, with an RHR >1 suggesting an overestimation in the subcohort, while an RHR <1 indicates an underestimation.

Both the general and subset analyses were adjusted for a comprehensive set of covariates to control for potential confounding. At the individual level, covariates included age (stratified into 5-year bands), sex (stratified), marital status, highest completed education, occupational status, individual wealth (tertiles), family income (tertiles), and household size. At the population level, adjustments were made for region (stratified into five administrative regions), parish-level population density, municipality-level access to healthcare (measured as the number of general practitioners per citizen), and parish-level SES, including mean income, median wealth, unemployment rate, and the rate of primary or low education. Additionally, we accounted for the SES difference between municipalities and parishes to capture relative inequalities in socioeconomic conditions. These covariates were selected a priori based on their established relevance to air pollution, health outcomes, and contextual importance for infectious diseases, ensuring robust control of potential confounding factors.

To quantify the uncertainty of the RHR, we employed bootstrapping with 200 resamples. This approach generated a distribution of RHR estimates, from which we derived the 95% CIs.

### Adjustment for collider bias

Following the methodological principle proposed by Thompson,^17^ we utilized external data from the Danish National Health Survey in 2017,^24^ and applied IPW to reweight the enrolled probability for participants in the patient-based cohort to obtain collider-bias-free estimates.

Specifically, we first employed the Danish National Health Survey data to estimate the probability of enrollment (i.e., the risk of hospitalization or SARS-CoV-2 infection) using conditional logistic regression models. This model conditioned enrollment probability on individuals’ exposure to air pollution and stratified by survival status. Once the models were constructed, we applied them to calculate the enrollment probability for each individual in the subcohort. The IPWs were then computed as the inverse of these probabilities.

To mitigate the influence of extreme weights, we truncated the IPWs at the 5^th^–95^th^ percentiles of their distribution. These weights were subsequently incorporated into weighted Cox proportional hazards models to estimate the association between air pollution and COVID-19 mortality, adjusted for individual- and area-level covariates.

Finally, to evaluate the effectiveness of the IPW approach, we repeated the procedure for quantifying bias on the bias-adjusted estimates. This allowed us to assess the extent to which the method successfully mitigated collider bias in our analysis.

RHR and HR for PM_2.5_ and NO_2_ were reported per 0.55 and 3.49 μg/m^3^ increase, respectively, which correspond to the interquartile range of PM_2.5_ and NO_2_ concentrations in the general population cohort.

## Results

The original cohort included 3,743,013 subjects, and our analysis included 3,721,810 participants (99.4% of the original population) after excluding individuals with missing information on SES or air pollution and with positive SARS-CoV-2 test before baseline. Compared with the total population (N = 3,721,810), subcohort I (SARS-CoV-2 infected, N = 138,742) was younger, had a higher proportion of employed individuals, and exhibited slightly higher SES, such as lower rates of low income and low wealth. In contrast, subcohort II (hospitalized patients, N = 11,270) was older, had a lower proportion of employed individuals, and exhibited lower SES, with higher rates of low income and lower rates of higher education (Table 1). COVID-19 mortality rates also varied significantly across the cohorts. In the total population, the mortality rate was 0.7‰ (2557 deaths), while it was substantially higher in subcohort I (18.4‰; 2549 deaths) and highest in subcohort II (165.8‰; 1869 deaths) (Table 1).

**Table 1.** Descriptive analysis for the general population and subpopulation in the AIRCODEN cohort at the study baseline on 1 March 2020 (n [%] or mean ± standard deviation [SD])

	Total population	SARS-CoV-2 infected	COVID-19 hospitalization
N	3,721,810	138,742	11,270
COVID-19 mortality, n (‰)	2,557 (0.7)	2,549 (18.4)^a^	1,869 (165.8)
Individual level			
Age, year (mean ± SD)	56.3 ± 15.6	52.0 ± 14.6	68.1 ± 15.6
Female, n (%)	1,904,171 (51.2)	72,250 (52.1)	5,024 (44.6)
Employed, n (%)	2,124,059 (57.1)	96,036 (69.2)	3,042 (27.0)
Married/partner, n (%)	2,069,552 (55.6)	84,220 (60.7)	6,019 (53.4)
Danish origin, n (%)	3,263,925 (87.7)	103,383 (74.5)	8,739 (77.5)
Higher education, n (%)	477,065 (12.8)	19,288 (13.9)	0,742 (6.6)
Low income, n (%)	1,082,427 (29.1)	38,286 (27.6)	5,266 (46.7)
Low wealth, n (%)	1,072,333 (28.8)	47,463 (34.2)	2,175 (19.3)
Family size ≤ 2, n (%)	3,313,067 (89.0)	117,557 (84.7)	9,856 (87.5)
Area level			
Mean income, DKK (mean ± SD)	287,915 ± 67,593	289,951 ± 74,259	287,678 ± 75,397
Median wealth, DKK (mean ± SD)	120,780 ± 169,063	110,713 ± 176,085	108,098 ± 176,085
Unemployment rate, % (mean ± SD)	1.0% ± 0.5	1.2% ± 0.5	1.2% ± 0.5
low education rate, % (mean ± SD)	22.6% ± 7.6	21.5% ± 7.6	22.3% ± 7.7
Population density, n/km^2^ (mean ± SD)	20.8 ± 42.3	30.2 ± 51.4	27.8 ± 45.8
GP visit rate, % (mean ± SD)	77.3% ± 2.0	76.8% ± 2.2	76.9% ± 2.1
Air pollution in 2019			
PM_2.5_, μg·m^−3^ (mean ± SD)	7.4 ± 0.5	7.5 ± 0.4	7.5 ± 0.4
NO_2_, μg·m^−3^ (mean ± SD)	10.7 ± 2.4	11.5 ± 2.4	11.4 ± 2.3

a Eight individuals were confirmed to be infected with SARS-CoV-2 by postmortem testing.

GP indicates general practice; NO_2_, nitrogen dioxide; PM_2.5_, particulate matter with diameter ≤2.5 μm.

The exposure of air pollution was slightly lower in the main cohort compared with the subcohort. The mean concentration levels of NO_2_ for main and subcohorts were about 10.7 and 11.5 µg/m^3^ with standard deviation around 2.4 µg/m^3^, and the mean concentration of PM_2.5_ was observed to be 7.4 and 7.5 µg/m^3^, with standard deviation around 0.4 µg/m^3^.

**Figure 2.**
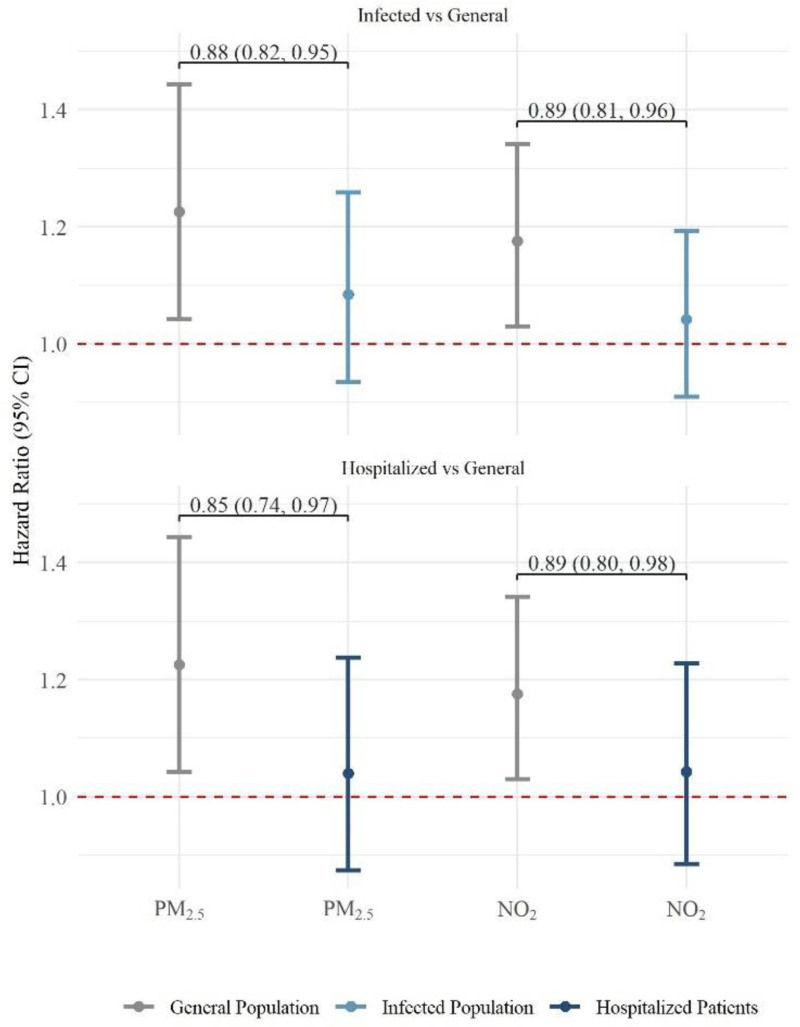
Relative hazard ratios based on adjusted hazard ratios for the association between long-term exposure to air pollution and COVID-19 mortality in the general and subpopulation in the AIRCODEN cohort. The association between long-term exposure to air pollution and COVID-19 mortality was presented as a hazard ratio (HR) with 95% confidence interval (95% CI). HR was estimated using a stratified Cox regression model, after adjusting for calendar time (time axis), sex (strata), age at baseline (strata), region (strata), marital status, household size, individual wealth, family income, education, occupational status, parish-level population density, mean income, median wealth, unemployment rate, primary or low education rate, the difference of those variables between parish and municipality, and municipality-level access to healthcare. HRs for the general population were obtained from previous studies. Results were presented as per interquartile range increase for each pollutant in the general population: 0.55 μg·m^−3^ for PM_2.5_, and 3.49 μg·m^−3^ for NO_2_.

We detected consistent and moderate effects of collider bias in estimating the association between long-term exposure to air pollution and COVID-19 mortality in subcohorts I and II where the base population was defined by the infected and hospitalized population, respectively (Figure 2). The RHRs (95% CI) for the risk of COVID-19 mortality were 0.88 (0.82, 0.95) and 0.85 (0.74, 0.97) per 0.55 µg/m^3^ increase in PM_2.5_ based on infected and hospitalized population, respectively, where the hazard ratios attenuated from 1.23 (1.04–1.44) in general population to 1.08 (0.93–1.26) and 1.04 (0.87–1.24) in subcohorts I and II, respectively. A similar attenuation of the associations was also observed with long-term exposure to NO_2_.

After applying the IPWs derived from the external source, we observed moderate increases in the estimated associations (Table 2). Specifically, the HR of per 0.55 µg/m^3^ increase in PM_2.5_ changed from 1.08 (0.93–1.26) and 1.04 (0.87–1.24) to 1.19 (1.05, 1.35) and 1.12 (0.95, 1.33) in subcohorts I and II, respectively. When compared with the estimates from general population, their corresponding RHRs were altered to 0.97 (0.84, 1.12) and 0.92 (0.75, 1.11), showing no significant bias. Changes were consistent for the associations with NO_2_, with even more pronounced shifts observed in correction for the SARS-CoV-2 infected population.

**Table 2.** The Collider-bias-adjusted association between long-term exposure to air pollution and COVID-19 mortality in the subpopulation in the AIRCODEN cohort

	Adjusted model	Adjusted model + IPW	Collider-bias-adjusted HR/HR from general population
	HR (95% CI)	HR (95% CI)	RHR (95% CI)
SARS-CoV-2 infected people			
PM_2.5_	1.08 (0.93, 1.26)	1.19 (1.05, 1.35)	0.97 (0.84, 1.12)
NO_2_	1.04 (0.91, 1.19)	1.39 (1.25, 1.55)	1.19 (1.05, 1.38)
Patient hospitalized with COVID-19			
PM_2.5_	1.04 (0.87, 1.24)	1.12 (0.95, 1.33)	0.92 (0.75, 1.11)
NO_2_	1.04 (0.88, 1.23)	1.33 (1.14, 1.55)	1.14 (0.99, 1.32)

The association between long-term exposure to air pollution and COVID-19 mortality was presented as a hazard ratio (HR) with 95% confidence interval (95% CI). HR was estimated using a stratified Cox regression model, after adjusting for calendar time (time axis), sex (strata), age at baseline (strata), region (strata), marital status, household size, individual wealth, family income, education, occupational status, parish-level population density, mean income, median wealth, unemployment rate, primary or low education rate, the difference of those variables between parish and municipality, and municipality-level access to healthcare. Collider-bias-adjusted HRs were further estimated by inverse probability weights (IPW), calculated from the external Danish National Health Survey. Results were presented as per interquartile range increase for each pollutant in the general population: 0.55 μg·m^−3^ for PM_2.5_, and 3.49 μg·m^−3^ for NO_2_.

## Discussion

We used register-based data to empirically study the consequence of using infected or hospitalized participants in the cohort design to examine the association between long-term exposure to air pollution and COVID-19 mortality. Our results indicated the associations are prone to be underestimated in patient-based cohorts due to potential collider bias and such bias seems to be stronger for association with PM_2.5_ when limited to hospitalized participants than the infected. We also showed that weighted analysis using IPWs derived from external data had the potential to account for such collider bias.

Given the increased evidence documenting the link between long-term exposure to air pollution, genetic factors, occupational exposure, social behaviors, and underlying health conditions (e.g., cardiovascular disease [CVD]) with an elevated risk of SARS-CoV-2 infection and subsequent hospitalization,^5–7^ restricting the analysis to infected or hospitalized patients may introduce collider bias. This bias arises because infection and hospitalization act as a collider: it is influenced by both air pollution (through its impact on disease severity) and other risk factors (e.g., CVD, occupational exposure, or social behaviors). Conditioning on hospitalization can create spurious associations or bias an existing one between air pollution and these risk factors. Take CVD as an example that has true associations with both air pollution and COVID-19 severity. In the general population, air pollution is well-documented to be positively associated with the increased risk of developing CVD.^25,26^ However, when conditioning on hospitalized COVID-19 patients, this association can weaken, disappear, or even reverse due to the overrepresentation of two subgroups: (1) individuals with CVD but low air pollution exposure, and (2) individuals with high air pollution exposure but no CVD. This distortion, in turn, may produce a biased estimation between air pollution and COVID-19 mortality.

**Figure 3.**
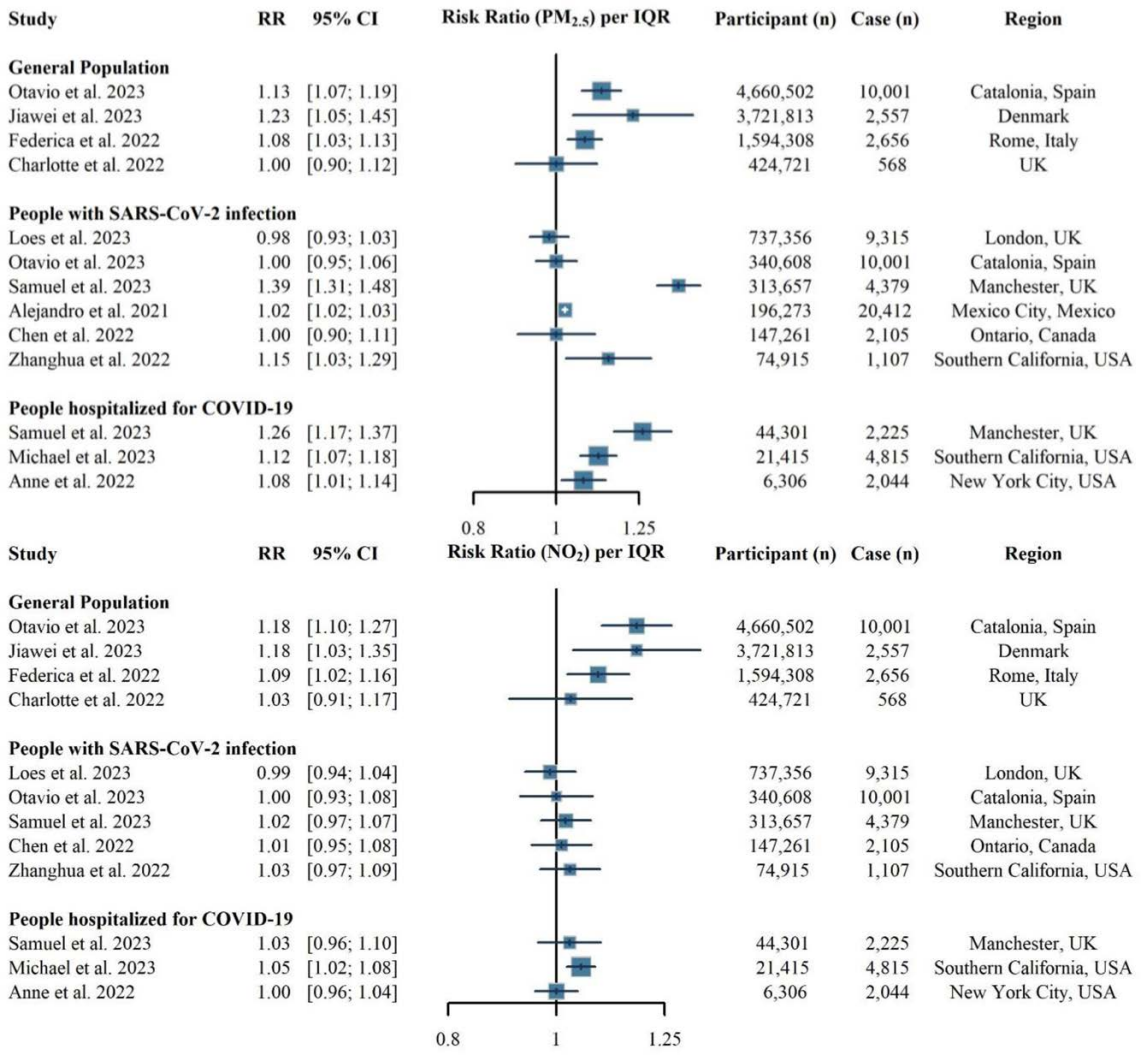
Summary of study on the association between COVID-19 mortality and long-term exposure to PM_2.5_ and NO_2_. Results from each study were presented as hazard ratio/odds ratio per study-specific interquartile range increase for each pollutant. The studies were presented by stratifying the underlying study population, including the general population, SARS-CoV-2 infected population, or COVID-19 hospitalized patients.

Our result provided empirical insights into the direction and magnitude of collider bias in estimating the association of long-term exposure to air pollution with COVID-19 mortality. These findings were particularly relevant for interpreting the current body of evidence. A recent systematic review highlighted inconsistencies and mixed results in studies evaluating this association, potentially due to collider bias.^5^ Our findings indicated that restricting analyses to populations either infected or hospitalized may skew estimates toward the null, aligning with trends observed in current studies (Figure 3), where patient-based cohorts often report null associations. Among the four studies conducted in the general population, one study from UK biobank-England,^27^ which was established a decade before the COVID-19 pandemic, reported null associations between air pollution (PM_2.5_ and NO_2_) and COVID-19 mortality. In contrast, three studies from Denmark,^19^ Spain,^28^ and Italy^29^ all reported consistent positive association of both PM_2.5_ and NO_2_. Among six studies focusing on SARS-CoV-2-infected individuals, three studies detected a positive association between PM_2.5_ and COVID-19 mortality,^30–32^ while none of these studies found an association with NO_2_ exposure.^28,31–34^ Evidence from studies limited to hospitalized COVID-19 patients was mixed. A study from Southern California reported a consistent positive association for both PM_2.5_ and NO_2_ with COVID-19 mortality,^35^ but two studies, from New York^36^ and Manchester,^31^ provided mixed findings, with inconsistent evidence for each pollutant.

Currently, only two studies have simultaneously explored the association between long-term exposure to air pollution and COVID-19 mortality using different baseline populations.^28,31^ In a Catalonia cohort with more than 4.6 million adults, Otavio et al.^28^ found that an increase of per 3 µg/m^3^ in PM_2.5_ or 16 µg/m^3^ in NO_2_ was associated with a 13%–18% increase in COVID-19 mortality risk in general population. However, this association attenuated to null when the analysis was limited to participants with SARS-CoV-2 infection.^28^ Similarly, in a Manchester cohort of 313,657 participants with SARS-CoV-2 infection, Samuel et al.^31^ reported an odds ratio of 1.39 for COVID-19 mortality associated with per 2 µg/m^3^ increase in PM_2.5_, which attenuated to 1.26 when the analysis was restricted to hospitalized patients. Our study corroborated Otavio et al.^28^’s findings, suggesting that such collider bias drives estimates toward the null. Furthermore, combining evidence from Samuel et al.,^31^ our study indicates a stronger bias when using hospitalized patients compared with infected. This underscores the necessity of accounting for collider bias in epidemiological studies to ensure accurate estimation of health risk factors.

Our findings further demonstrated that adjusting for collider bias using IPW resulted in moderate increases in the estimated associations between air pollution (PM_2.5_ and NO_2_) and COVID-19 mortality. These results align with previous studies highlighting the role of air pollution in contributing to COVID-19 mortality and represent a novel approach to addressing collider bias in environmental epidemiology research. This approach is particularly valuable in research settings where direct access to general population data is not feasible, as it enables researchers to leverage data from hospitalized patients while adjusting for collider bias using publicly available or external datasets. Several alternative approaches have also been proposed and implemented in the previous literature to bound bias. For example, a negative control design^37^ offers a useful tool to detect the presence of unmeasured confounding or selection bias. This involves testing the association using negative exposure control or negative outcome control, which are presumed not to influence the outcome or not affected by the exposure. Furthermore, when representative sampling data is not available, quantitative bias analysis could be another approach.^38^ Through this, researchers could use the estimated RHR provided here, along with the external data or literature, to specify plausible bias parameters for selection mechanisms, and explore what the true exposure–outcome association would be in the absence of collider bias.^11,38^

However, it is also important to acknowledge that methods to account for collider bias remain limited,^6^ particularly in research settings where only collider-samples (e.g., hospitalized patients) are available. In such cases, it is not feasible to fully adjust for collider bias without additional data from the general population.^6^ Previous studies with patient-based cohorts have attempted to account for collider bias by adjusting for contextual factors such as population density and area information in access to healthcare,^34^ our findings suggest that this approach has limitations. The persistence of a significant RHR after adjusting for these variables indicates that residual confounding remains, likely due to unmeasured risk factors and population differences. For example, individual-level social behaviors, occupational exposures, or genetic predispositions—which were not captured in our analysis—may still influence both the likelihood of infection and the severity of outcomes. These unmeasured risk factors, particularly those that are consequences of the exposure (e.g., air pollution), could contribute to the observed associations. Therefore, the significant RHR after adjustment serves as evidence that the methods used to control for collider bias are insufficient, highlighting the need for more robust approaches to fully address this issue.

This study benefited from the unselected large population samples through register linkage, which enables us a nuanced exploration and comparison of collider bias in the real-world setting. However, several limitations should also be further noticed. First, RHR method is built on comparing the biased estimate with the potential unbiased estimate. When estimating the associations from the general population, we have tried to control for comprehensive individual- and area-level indicators. As a register-based study, potential residual confounding remains, especially since we lacked individual lifestyle and behavioral factors, such as smoking, physical activities, mask mearing, and social contacts, in our dataset. However, previous studies have documented that the influence of further adjusting smoking and physical activity is limited in estimating the association between air pollution and COVID-19 mortality in both the general population^28^ and infected individual setting.^31^ Second, we modeled the long-term exposure to air pollution at residual address levels after accounting for the change of residual address, but we lacked exposure data on workplace and commuting, leaving us a potential exposure misclassification. While such bias would result in lower precision in HR estimates and share similar causal structures under general and subsample, its influence on RHR would be limited. Third, while IPW provides a robust framework for addressing selection bias, its effectiveness depends on the representativeness of the external data and the accuracy of the enrollment probability model.^17^ The significant RHR can still be seen for the association of NO_2_ in SARS-CoV-2 infected sample after applying IPWs, suggesting residual selection bias even after weighting. One possible explanation is the strong urban–rural disparity in NO_2_ exposure, which may be more strongly correlated with nonresponse patterns in survey-based samples. Prior research^24^ has shown that individuals from urban areas with higher SES are more likely to be included in such samples, leading to differential inclusion probabilities. This correlation between exposure and selection mechanisms could introduce collider bias, particularly for NO_2_, and may not be fully addressed by the IPW model. This highlights the complexity of correcting for selection bias in environmental exposure studies and underscores the need for caution when interpreting pollutant-specific associations derived from selected populations. Finally, as highlighted in previous studies,^39,40^ conducting epidemiological research on infectious diseases presents significant challenges, including issues related to case ascertainment, vaccination strategies, spatiotemporal variation of viral circulation, and various forms of bias. These complexities can substantially influence study findings and interpretations. For instance, the definition of COVID-19 mortality varies across countries and studies, with some using COVID-19-specific deaths, obtaining from death certificates, while others, including our study, define it as all-cause mortality within 30 days of infection. Differences in outcome definitions can influence absolute effect estimates and comparability across studies. However, evidence from the UK^41^ and Denmark^42^ indicates that these definitions largely aligned during our study period, with over 90% agreement between the two measures before the emergence of the Omicron variant and widespread booster vaccinations, which occurred after our follow-up period. While addressing all potential bias goes beyond the scope of our current study, we focus on collider bias—a specific methodological issue that has received comparatively less attention, and aims to contribute to a more nuanced understanding of how selection mechanisms can distort observed associations, providing valuable insights for future epidemiological research.

In conclusion, our study indicated that restricting the study population to infected or hospitalized patients would introduce collider bias into the study. Such bias would result in an underestimation of the association when the association between exposure and outcome and the association between exposure and selection are both positive or negative. We further proposed and demonstrated that IPWs could be a useful tool for epidemiologists to account for such bias. This is particularly relevant for current evidence on air pollution and COVID-19. In this context, we underscored the caution for researchers analyzing infected or hospitalized patient data, especially when interpreting null associations in patient-based cohort studies. Although options to mitigate such biases are limited beyond study design, the quantification of bias parameters and the application of IPWs in our study may provide valuable baseline data for future research endeavors. By comparing our estimates with those derived from different populations or healthcare systems, researchers can better understand how collider bias varies across contexts and refine methodologies for addressing it.

The study was conceptualized and designed by J.Z. and Y.-H.L. Statistical analysis was conducted by J.Z. Z.J.A. is the PI of the AIRCODEN cohort. All authors have read and revised the manuscript and contributed to the interpretation of the results. All authors have approved the final draft of the manuscript.

## Conflicts of interest statement

The authors declare that they have no conflicts of interest with regard to the content of this report.
